# Reciprocal vs nonreciprocal trade agreements: Which have been best to promote exports?

**DOI:** 10.1371/journal.pone.0210446

**Published:** 2019-02-04

**Authors:** Salvador Gil-Pareja, Rafael Llorca-Vivero, José Antonio Martínez-Serrano

**Affiliations:** Department of Applied Economics II, University of Valencia, Valencia, Spain; Universidad de Castilla-La Mancha, SPAIN

## Abstract

The Doha Development Agenda recognizes the central role that international trade can play in the promotion of economic development. In fact, the increase of exports from developing countries to developed nations' markets has been considered a key element for developing countries to realize the potential benefits of globalization. Over the last decades, developed countries have provided preferential access to their markets to developing countries through nonreciprocal trade agreements. Moreover, developing countries have also participated in reciprocal trade agreements. This paper re-examines comparatively the effect of both kinds of trade agreements on exports from developing countries but also from the developed world. In line with other studies, our results across specifications are unstable. However, the results of our preferred specification give additional support to the argument raised by critics of nonreciprocal preference regimes who consider that developing countries should abandon their reliance on one-way trade preferences in favor of reciprocal agreements.

## 1. Introduction

One of the most important challenges in the Doha’s round of trade negotiations is the promotion of development. The Doha Development Agenda recognizes the central role that international trade can play in the promotion of economic development. In fact, the rise of exports from developing countries to developed nations' markets has been for a long time viewed as a key element for developing countries to realize the potential benefits of globalization. The usual approach to accomplish this aim has implied that the developed world give support to the integration of developing countries into their economies through a series of unilateral concessional measures towards developing countries in the form of nonreciprocal trade preferences. In addition to all developed countries, since 2000s a number of emerging economies (Morocco, Turkey, India, China, Russia, Chile or Thailand, among others) also offer their own programs focused on Least Developed Countries (LDCs), although they are typically limited in scope. [1] provides the list of existing programs including their starting year, the current number of beneficiaries, their key features and the main online source of information for each program of preferential access.

The main instrument for the so-called “special and differential treatment” for developing countries has been the Generalized System of Preferences (GSP), but there have been many other nonreciprocal preferential trade agreements (NRPTAs). Examples of these others nonreciprocal programs are several EU and USA initiatives (the GSP+, ACP-EU partnership agreement, the Everything but Arms arrangement, the Caribbean Basin Initiative, the Andean Trade Preference Act and the African Growth and Opportunity Act), or the duty-free treatment granted by many other countries for least developed countries or for developing countries from a specific region. The intellectual underpinnings for the “special and differential treatment” arrangements in GATT for developing countries go back to the 1950s. They were based on balance of payments problems for developing countries, protection on infant-industry grounds and the Singer-Prebisch thesis about the secular decline in developing countries terms of trade ([2]).

As is well known, trade arrangements for developing countries have not been confined to one-way trade preferences. On the one hand, a large number of developing countries are members of the GATT/WTO system, which is based on the reciprocity and the most-favored nation principles. On the other hand, developing countries have also made a conscious effort to forge reciprocal preferential trade agreements, involving only developing countries (known as South-South agreements) or implicating both developing and developed countries (known as North-South agreements). The Common Market of Eastern and Southern Africa (COMESA), the Association of Southeast Asian Nations (ASEAN), and the *Mercado Común del Sur* (MERCOSUR) are examples of South-South agreements. In contrast, the North American Free Trade Agreement (NAFTA), the agreement between Canada and Chile and that between the European Union and Mexico are examples of North-South agreements.

Critics of nonreciprocal preference schemes have traditionally argued that developing countries should abandon their reliance on one-way trade preferences in favor of reciprocal agreements, since the latter implies a stronger, credible and lasting commitment (see, for example, [3]; [4] and [5]). This approach is also advocated by those who believe that the infant-industry argument, often used to justify unilateral concessions, is a fallacious argument.

The gravity equation has become the main econometric approach for examining *ex post* the “partial” (or direct) effects of economic integration agreements on aggregate bilateral trade flows. After accounting for multilateral resistance terms with time-varying fixed-effects and controlling for endogeneity bias using panel data techniques, [6] find that free trade agreements do increase countries’ bilateral trade flows significantly using data at five-year intervals from 1960 to 2000 for 96 countries. Following this empirical strategy and the same data set, [7] go a step further by comparing the impacts of North-South and South-South trade agreements on bilateral trade and show that free trade agreements lead to an increase in bilateral trade regardless of whether the signatories are developing or developed countries. In particular, they find that the percentage rise in bilateral trade is higher for South-South agreements that for North-South agreements.

Moreover, other recent studies focus on investigating the impact of nonreciprocal trade agreements on bilateral trade including also controls for regional trade agreements. [8] analyse the GSP with annual data for 184 countries over the period 1953–2006 and find that the GSP tends to foster exports in both directions in the short-run, but hampers them in the long-run. In contrast, [9] and [10], with data for 177 countries over the period 1960–2008, provide an in-depth analysis on the issue finding robust evidence that NRPTAs positively affect developing countries’ exports to developed countries but also find a positive effect in the opposite direction, that is, from benefactor countries to beneficiary countries. [11] examine the effects of various types of economic integration agreements (including one-way preferential trade agreements) on trade flows and trade margins with data at five-year intervals from 1965 to 2000, concluding that deeper integration agreements have larger impacts on aggregate trade flows, extensive margins and intensive margins than shallower agreements. [12] study the extent to which developing countries export more as a result of being in the official LDCs list and the effect of unilateral preference regimes. Using data over the period 1970–2013 they find that the inclusion in the LCDs list is associated with substantially higher exports. However, unilateral preference regimes are, on average, not always beneficial in terms of exports for beneficiary developing countries but do have some impact in some sectors. Finally, [13], with data between 1960 and 2015, find that whereas the average trade effect of the nonreciprocal preferences on the exports of beneficiaries is highly unstable across specifications the effect is strong and robust when these countries are members of the WTO and very poor. In contrast, non-LDCs beneficiaries only expand exports if they are not WTO members.

This paper re-examines comparatively the impact on exports from developing countries to developed countries of both reciprocal and nonreciprocal trade agreements using the latest available data and techniques of structural gravity estimation and taking into account the direction of export flows of the reciprocal agreements. The use of data up to 2016 allows us to analyze whether the nonreciprocal preferences have been eroded in recent years due to the numerous reciprocal trade agreements signed between developed and developing countries over the last decade. The sample period also incorporates the revision of the European GSP in 2014, when several middle income countries lost their preferential access to the European Union. Moreover, in line with [10] results, we also examine the potential differential impact of both types of agreements on exports from developed countries to beneficiary countries. From an econometric point of view, we control simultaneously for several sources of bias (multilateral resistance terms, unobserved bilateral heterogeneity, heteroskedastic residuals, and zero trade flows), which is in line with current “state of the art” in the literature on the gravity equation.

To preview our results, we find that the evidence reported varies across specifications. However, according to our preferred specification, PPML estimation with the dependent variable in export shares and allowing for correlation in the error term within all possible cluster dimensions, for the whole sample period only reciprocal agreements have had a positive effect in trade flows between developed and developing countries and only when the exporter is the developing country. For data up to 2008, there is evidence of a positive impact of the NRPTA on exports from beneficiary countries that vanishes in the last years. Finally, for exports from developed countries to beneficiary countries our preferred specification does not provide evidence of a significant effect in any case.

The paper is organised as follows. Section 2 presents the methodology. Section 3 describes the data. Section 4 discusses the estimation results. Finally, section 5 concludes the paper.

## 2. Methodology

Since it was independently developed by [14]and [15] more than five decades ago, the gravity model has become the key econometric framework for estimating *ex post* the “partial” (or direct) effects of different kinds of economic integration agreements on bilateral trade flows. Our estimation strategy follows that of [6] and [16]. In particular, we control for multilateral resistance terms by including exporter-time and importer-time fixed effects. [17] emphasized that the gravity model theory implies that it is not just bilateral trade costs (the bilateral resistance to trade), but also the trade costs relative to the rest of the world (the multilateral resistance to trade) that are relevant for predicting bilateral trade flows. Moreover, we control for endogeneity by means of bilateral fixed effects. This issue has received a great deal of attention in the empirical gravity-equation literature since [6] noted that trade agreements are not exogenous. They showed that *ex post* estimation of the partial effects of free trade agreements (FTA) suffered from endogeneity bias, mainly due to self-selection of country-pairs into agreements (as a result of pre-existing trade levels), and find that this self-selection bias may be substantially reduced when employing pair-specific fixed effects or using first-differenced data.

As noted by [6] a few papers attempted to deal with the potential bias caused by endogenous PTAs in cross-section gravity equations with instrumental variables providing mixed and unstable results (see, for instance, [18] or [19]). Moreover, it is worth noting that finding appropriate instruments is not an easy task. The approach of [6] assumes that the main source of bilateral bias is time invariant and so they argue that panel regression techniques (estimation with country-pair fixed effects or first differencing) are more suitable to account for endogeneity and yield more stable results. An alternative way to deal with endogeneity is to use matching techniques. Obviously, country-pair fixed effects do not fully eliminate the concern about potential selection bias (endogeneity) since countries could adopt a PTA after a surge in trade within the sample period.

Since the late 2000s, empirical work on the determinants of trade flows has increasingly relied on a theoretically motivated gravity equation that controls for both multilateral resistance terms and unobserved bilateral heterogeneity with three sets of fixed effects: exporter-time, importer-time and country-pair (see, for example, [20], [21], [22], [23], [24],[25]-, [26], [7], [27] or [28]).

Our benchmark specification is the gravity Eq (1), which comprehensively accounts for multilateral resistance terms by including time-varying fixed effects and for self-selection endogeneity bias with country-pair fixed effects:

$$\ln X_{ijt}=\beta_{0}+\beta_{1}CU_{ijt}+\beta_{2}PTA_{ijt}+\beta_{3}GATT/WTO_{ijt}+\beta_{4}NRPTAXbenMdev_{ijt}+\beta_{5}NRPTAXdevMben_{ijt}+\eta_{ij}+\chi_{it}+\lambda_{jt}+u_{ijt}$$ (1)

where *i* and *j* denote trading partners, *t* is time, and the variables are defined as follows: *X*_*ijt*_ are the bilateral export flows from *i* to *j* in year *t*, *CU*, *PTA*, *GATT/WTO* are binary variables for common membership in currency unions, preferential trade agreements and General Agreement on Tariffs and Trade/World Trade Organization, *NRPTAXbenMdev*_*ijt*_ (*NRPTAXdevMben*_*ijt*_) is a binary variable which is unity if *i* is a beneficiary (benefactor) of a nonreciprocal preferential trade agreement and *j* is the corresponding preference-giving (beneficiary) country, η_ij_ are country-pair fixed effects, χ_it_ and λ_jt_ are exporter-year and importer-year fixed effects, respectively, and *u*_*ijt*_ is the standard classical error term.

The benchmark specification includes only one dummy for all (reciprocal) preferential trade agreements (PTA). In order to investigate the issues addressed in this paper, we disaggregate the dummy variable PTA in three different ways by interacting the PTA dummy with dummies for whether the exporter and/or the importer countries are or not beneficiaries of nonreciprocal preferential trade agreements. Firstly, we split this dummy into two dummies depending on whether the exporter is a beneficiary country (*PTAXben*) or a developed country (*PTAXdev*). Secondly, we split the PTA dummy into two from the importer’s perspectives (denoted by *PTAMben* and *PTAMdev*). Finally, and most importantly, we split the PTA dummy into four dummies taking into account the group which each trading partner in the pair belong to: Exporter beneficiary and importer developed (*PTAXbenMdev*), exporter developed and importer beneficiary (*PTAXdevMben*), exporter and importer beneficiary (*PTAXbenMben*) and exporter and importer developed (*PTAXdevMdev*). For further clarification, let consider the first of these dummies as an example. *PTAXbenMdev* is a dummy that is unity if country *i* is a beneficiary country of a nonreciprocal trade agreement and country *j* is a developed country and they share membership in a reciprocal preferential trade agreement and zero otherwise. Comparing the estimated coefficient for this variable with that obtained for *NRPTAXbenMdev* allows us to test whether reciprocal or nonreciprocal trade agreements have been best to promote exports from developing countries. In a similar way, we can compare the export performance of developed countries participating in reciprocal (*PTAXdevMben*) and nonreciprocal (*NRPTAXdevMben*) trade agreements in trade with beneficiary countries.

Moreover, in line with other more recent works, we go further than [6] because we additionally account for heteroskedastic residuals and zero trade flows by estimating the gravity equation in levels rather than in logs with the Poisson Pseudo-Maximum Likelihood (PPML) estimator. This estimator was popularized by [29] to deal with a potential bias related to heteroscedasticity arising through log-linearization of gravity equations, but it is also useful by its ability to handle zeros in bilateral trade flows. After [29], a large number of recent papers deals with both econometric problems using PPML (see, for example, [30], [31], [32], [33], [34], [9], [28], [10]). However, the large datasets used in most of these studies or difficulties to achieve convergence have precluded to estimate the Poisson gravity equations including, at the same time, the three types of high dimensional fixed effects that are required to account for both unobserved bilateral heterogeneity and multilateral resistance terms (country-pair fixed effects, exporter-time and importer-time fixed effects). Fortunately, [16] have recently implemented an iterative PPML estimator that allows researchers to control simultaneously for both issues in a large dataset like the one used in this paper. Taking advantage of this technical development, we estimate the following gravity equation with the PPML estimator:

$$X_{ijt}=\exp(\beta_{1}CU_{ijt}+\beta_{2}PTA_{ijt}+\beta_{3}GATT/WTO_{ijt}+\beta_{4}NRPTAXbenMdev_{ijt}+\beta_{5}NRPTAXdevMben_{ijt}+\eta_{ij}+\chi_{it}+\lambda_{jt})+u_{ijt}$$ (2)

## 3. Data

Data on the dependent variable (bilateral export flows) come from the Direction of Trade dataset (IMF). The sample covers 182 countries and territories over 15 years of the period 1960–2016 at four-year intervals. Data for currency unions are taken from the CIA's World Factbook. The indicators of preferential trade agreements and GATT/WTO have been built using data from the World Trade Organization. In this study, we use the expression “preferential trade agreement” to refer also to other agreements involving a higher degree of economic integration. In fact, most economic integration agreements considered in the sample are free trade agreements. Data on the one-way trade preferences come from different sources. Data on the African Growth and Opportunity Act and Everything but Arms initiative come from the corresponding websites. The list of beneficiaries of the Caribbean Basin Initiative and the Andean Trade Preference Act come from the Office of the United States Trade Representative. The listing of beneficiaries of the Cotonou Agreement (ACP-EU Partnership Agreement) comes from its website and[35]. The list of countries that are beneficiaries of the standard GSP programs are taken from the United Nations Conference on Trade and Development (UNCTAD). For years before 2000, we use data from UNCTAD kindly provided by Bernard Herz and Marco Wagner. Finally, the information on developing countries that provide their own programs for LDCs (Morocco, Turkey, India, China, etc.) comes from the WTO database on Preferential Trade Arrangements and from the websites of the individual programs. Tables A and B in S1 Appendix show some descriptive statistics.

The list of developed countries includes those countries that have never been beneficiaries of one-way trade preferences Australia, Austria, Belgium-Luxembourg, Canada, Denmark, Finland, France, Germany, Greece, Iceland, Ireland, Israel, Italy, Japan, the Netherlands, New Zealand, Norway, Portugal, South Korea, Spain, Switzerland, Sweden, the United Kingdom and the United States, plus Cyprus, Czech Republic, Estonia, Hungary, Latvia, Lithuania, Malta, Poland, Slovak Republic and Slovenia (after their accession to the European Union in 2004), and Bulgaria and Romania (after their accession to the European Union in 2007).

## 4. Empirical results

The estimation results for the structural gravity Eq (1) by OLS appear in column 1 of Table 1. As it is usual in the literature, we report standard errors clustered by country-pairs (in parentheses). In this specification, all time-invariant standard regressors of the gravity equation (such as the bilateral distance between countries or the use of a common language) are absorbed into the pair-specific fixed effects. Moreover, all exporter-specific and importer-specific time-variant variables (such as the GDPs or the theoretical multilateral resistance terms) are controlled for the time-varying country-year fixed effects for both exporters and importers. Before discussing the results, note that this is a linear (in logs) regression and, therefore, do not permit the inclusion of zeros and, most importantly, it may provide inconsistent parameter estimates due to the likely presence of heteroscedasticity in trade data ([29] and [36]). As it is usual, the gravity equation works well explaining a large percentage of the total variation of bilateral exports flows. Moreover, with the exception of the point estimate of the dummy for currency union, all estimated coefficients for the economic integration agreements are positive and statistically significant at conventional levels. The GATT/WTO dummy shows the largest point estimate (0.543) and the difference with respect the other estimated coefficients is statistically significant at conventional levels.

**Table 1:** Estimation results of the log-linear gravity equation. Sample period 1960–2016 at four-year intervals.

	(1)	(2)	(3)	(4)	(5)	(6)	(7)	(8)
CU	-0.067 (0.079)	-0.068 (0.079)	-0.077 (0.079)	-0.119 (0.082)	-0.052 (0.079)	-0.046 (0.079)	-0.145 (0.079)*	-0.094 (0.080)
PTA	0.236 (0.033)***				0.232 (0.033)***	0.231 (0.033)***	0.189 (0.032)***	0.236 (0.033)***
PTAXben		0.226 (0.038)***						
PTAXdev		0.251 (0.054)***						
PTAMben			0.189 (0.036)***					
PTAMdev			0.316 (0.059)***					
PTAXbenMdev				0.216 (0.056)***				
PTAXdevMben				0.111 (0.049)**				
PTAXbenMben				0.248 (0.049)***				
PTAXdevMdev				0.398 (0.084)***				
GATT/WTO	0.543 (0.048)***	0.542 (0.048)***	0.539 (0.048)***	0.548 (0.048)***				0.542 (0.048)***
GATTXben					0.441 (0.050)***			
GATTXdev					0.723 (0.057)***			
GATTMben						0.402 (0.050)***		
GATTMdev						0.800 (0.063)***		
GATTXbenMdev							0.620 (0.063)***	
GATTXdevMben							0.511 (0.057)***	
GATTXbenMben							0.332 (0.052)***	
GATTXdevMdev							1.710 (0.087)***	
NRPTAXbenMdev	0.167 (0.040)***	0.166 (0.040)***	0.183 (0.041)***	0.181 (0.041)***	0.166 (0.040)***	0.144 (0.040)***	0.178 (0.040)***	
NRPTAXdevMben	0.228 (0.033)***	0.231 (0.033)***	0.226 (0.032)***	0.227 (0.033)***	0.209 (0.032)***	0.229 (0.032)***	0.245 (0.032)***	0.222 (0.033)***
NRPTAXbenMdev (up to 2008)								0.239 (0.040)***
NRPTAXbenMdev (from 2008)								0.001 (0.050)
CYFE	Yes	Yes	Yes	Yes	Yes	Yes	Yes	Yes
CPFE	Yes	Yes	Yes	Yes	Yes	Yes	Yes	Yes
No observat.	170,295	170,295	170,295	170,295	170,295	170,295	170,295	170,295
Adj R^2^	0.89	0.89	0.89	0.89	0.89	0.89	0.89	0.89

Notes: We employ the *reghdfe* Stata command. The regressand is the log of bilateral exports. CU, PTA and NRPTA denote currency unions, preferential trade agreements and nonreciprocal preferential trade agreements, respectively. The suffixes Xben and Xdev in the name of the variables indicate exporter beneficiary and exporter developed, respectively. In a similar way, the suffixes Mben and Mdev in the name of the variables indicate importer beneficiary and importer developed, respectively. Robust standard errors (clustered by country-pairs) are in parentheses.

* significant at 10%

** significant at 5%

*** significant at 1%. CYFE indicates time-varying exporter and importer fixed effects. CPFE indicates country-pair fixed effects. Coefficient estimates for CYFE and CPFE are not reported for brevity.

Column 2 and 3 report the results when we split the PTA dummy from the exporter and the importer perspectives, respectively. In the first case, there are no statistical differences in the impact of PTAs depending on whether the exporter is a beneficiary or a developed country (regardless the group that the trading partner belongs to). In particular, coefficient estimates are close (0.226 for PTAs in which the exporter is a beneficiary country and 0.251 for PTAs in which the exporter is a developed country). In contrast, when the PTA dummy is split from the importer perspective, the estimated coefficient is positive in both cases but significantly higher (at the 5 per cent level of significance) when the importer is a developed country (0.316) than when it is a beneficiary country (0.189).

The next natural step is to split the PTA dummy taking into account the group to which each trading partner belong (column 4). The results suggest that preferential trade agreements have a positive effect in the four cases. The largest effect appears for trade flows between developed countries (0.3984) whereas the point estimates for the other three combinations goes in a range from 0.111 to 0.248.

With regard to the comparison between reciprocal (PTAs) and nonreciprocal agreements, these results reveal that for exports from beneficiary countries to developed countries both dummies are positive and despite the point estimate is larger for reciprocal agreements than for nonreciprocal the difference between them is not statistically significant. However, for exports from developed countries to beneficiary countries the impact is higher for nonreciprocal agreements.

Until now, the comparison between reciprocal and nonreciprocal trade agreements is based on PTAs. However, as noted before the multilateral trade liberalization under the auspices of the GATT/WTO is also reciprocal in nature. The empirical evidence about the impact of the GATT/WTO across groups of countries (developed versus developing) is varied ([37], [31], [23],[26], [38] and [28]). The empirical results go from negative impacts for developing countries to positive effects for both groups of countries.

Columns 5 to 7 of Table 1 presents the results when we repeat the regressions in columns 2 to 4 but disaggregating in this case the GATT/WTO dummy following the same procedure used with the variable PTA. When we split this variable from the exporter perspective (column 5) we find a statistically larger effect on trade for exports from developed countries than for exports from beneficiary countries. The same picture emerges from the importers perspective (column 6) suggesting that GATT/WTO membership promotes trade strongly between developed countries. In fact, when we further disaggregate the GATT dummy taking into account the group to which exporters and importers belong, we find that the largest estimated coefficient appears for trade between developed country members and the smallest for trade between beneficiary countries (column 7). In short, the GATT/WTO impact on trade across groups of countries is uneven but positive and statistically significant at the 1% level in all cases.

Comparing the estimated coefficients for the GATT/WTO and the nonreciprocal trade agreements we find that for both exports from beneficiary countries to developed countries and exports in the opposite direction the impact is larger for the GATT/WTO than for the nonreciprocal agreements, and the difference between the estimated coefficients is statistically significant at the 1 per cent level).

Finally, in column 8 we present the results when we split the NRPTA dummy for exports from beneficiary countries to developed countries into two: one for the years until 2008 and the other for data from 2008. This allows us to check whether the nonreciprocal preferences have been eroded in recent years, as a result of the proliferation of reciprocal PTAs between developed and developing countries over the last decade or the important revision of the European GSP that took place in 2014. The results confirm the erosion of preferences in the later years. The estimated coefficient for that variable from 2008 is very close to zero and not statistically significant at conventional levels. [12], when comparing the period 1970–2013 with the period that leaves out the last five years of their sample (1973–2008), also find evidence of partial erosion for the trade preferences granted by Canada and of total erosion in the cases of the trade preferences granted by Australia and the European Union.

As noted before, Table 1 reports standard errors clustered at the pair level only. In a recent paper, [39] investigate the consequences of disregarding the interdependence of the disturbances in multiple dimensions with cross-sectional and panel-data structural gravity models of bilateral trade when making inference. These authors conclude that ignoring multi-way clustering leads to misleading inference regarding the relevance of preferential trade-agreement memberships of different kinds, since multi-level clustering has large effects on the standard errors of trade-cost variables. To deal with this concern, Table 2 presents the results accounting for multi-level clustering, which allows for correlation in the error term within all possible cluster dimensions. Obviously, clustering does not affect the point estimates. The results show that despite there are substantial differences between both types of standard errors (multi-way clustered standard errors are higher) all the positive point estimates remain statistically significant at the 1 per cent level of significance, remaining unaltered all previous conclusions.

**Table 2:** Estimation results of the log-linear gravity equation. Sample period 1960–2016 at four-year intervals. Multi-way clustering estimation of standard errors.

	(1)	(2)	(3)	(4)	(5)	(6)	(7)	(8)
CU	-0.067 (0.110)	-0.068 (0.110)	-0.077 (0.110)	-0.119 (0.112)	-0.052 (0.109)	-0.046 (0.109)	-0.145 (0.109)	-0.094 (0.110)
PTA	0.236 (0.054)***				0.232 (0.053)***	0.231 (0.054)***	0.189 (0.050)***	0.236 (0.054)***
PTAXben		0.226 (0.057)***						
PTAXdev		0.251 (0.093)***						
PTAMben			0.189 (0.054)***					
PTAMdev			0.316 (0.092)***					
PTAXbenMdev				0.216 (0.086)**				
PTAXdevMben				0.111 (0.082)				
PTAXbenMben				0.248 (0.071)***				
PTAXdevMdev				0.398 (0.084)***				
GATT/WTO	0.543 (0.096)***	0.542 (0.095)***	0.539 (0.095)***	0.548 (0.093)***				0.542 (0.095)***
GATTXben					0.441 (0.093)***			
GATTXdev					0.723 (0.120)***			
GATTMben						0.402 (0.091)***		
GATTMdev						0.800 (0.121)***		
GATTXbenMdev							0.620 (0.112)***	
GATTXdevMben							0.511 (0.108)***	
GATTXbenMben							0.332 (0.087)***	
GATTXdevMdev							1.710 (0.185)***	
NRPTAXbenMdev	0.167 (0.060)***	0.166 (0.060)***	0.183 (0.062)***	0.181 (0.061)***	0.166 (0.060)***	0.144 (0.059)**	0.178 (0.059)***	
NRPTAXdevMben	0.228 (0.054)***	0.231 (0.056)***	0.226 (0.054)***	0.227 (0.056)***	0.209 (0.052)***	0.229 (0.054)***	0.245 (0.052)***	0.222 (0.056)***
NRPTAXbenMdev (up to 2008)								0.239 (0.066)***
NRPTAXbenMdev (from 2008)								0.001 (0.089)
CYFE	Yes	Yes	Yes	Yes	Yes	Yes	Yes	Yes
CPFE	Yes	Yes	Yes	Yes	Yes	Yes	Yes	Yes
No observat.	170,295	170,295	170,295	170,295	170,295	170,295	170,295	170,295
Adj R^2^	0.89	0.89	0.89	0.89	0.89	0.89	0.89	0.89

Notes: We employ the *reghdfe* Stata command. The regressand is the log of bilateral exports. The regressand is the log of bilateral exports. CU, PTA and NRPTA denote currency unions, preferential trade agreements and nonreciprocal preferential trade agreements, respectively. The suffixes Xben and Xdev in the name of the variables indicate exporter beneficiary and exporter developed, respectively. In a similar way, the suffixes Mben and Mdev in the name of the variables indicate importer beneficiary and importer developed, respectively. Robust standard errors (multi-way clustering) are in parentheses.

* significant at 10%

** significant at 5%

*** significant at 1%. CYFE indicates time-varying exporter and importer fixed effects. CPFE indicates country-pair fixed effects. Coefficient estimates for CYFE and CPFE are not reported for brevity.

Moreover, it is important to deal with the econometric problems resulting from both heteroskedasticity and the existence of zero values in bilateral trade flows. It allows us to avoid other potential sources of bias in the estimation that could be present in the log-linear specification. This paper benefits from the recent computational development implemented by [16], which makes feasible to run the PPML estimator including the three types of high dimensional fixed effects required to get unbiased, and theory-consistent estimates. Column 1 of Table 3 shows the estimates of gravity Eq (2) with PPML. To save on space, under each coefficient we report the two types of standard errors: clustered by country-pair (in parentheses) and multi-way clustered standard errors (in brackets). Comparing these results with those offered in column 1 of Table 1 we can see that switching from OLS to PPML leads to a loss of the statistical significance of all the variables. At this point, it is worth noting that the loss of significance of the estimated coefficients of both PTAs and GATT variables, when we switch from OLS to PPML, is consistent with the result found by [40]. Moreover, these authors, as previously did [16], also show insignificant result regarding the trade effect of the euro when using PPML but not when using OLS (including high-dimensional fixed effects in both cases). However, a remarkable difference between [16] and [40] papers when using OLS is that the former finds a positive and significant effect for the euro, while the later finds a negative and significant impact.

**Table 3:** PPML results. Data at four-year intervals.

	(1)	(2)	(3)	(4)	(5)	(6)	(7)
Sample period	1960–2016	1960–2016	1960–2016	1960–2016	1960–2016	1960–2008	1960–2008
Dep. variable	exports	export share	export share	export share	export share	export share	export share
CU	0.018 (0.040) [0.054]	0.428 (0.075)*** [0.128]***	0.422 (0.075)*** [0.128]***	0.415 (0.075)*** [0.128]***	0.419 (0.075)*** [0.127]***	0.413 (0.074)*** [0.128]***	0.407 (0.092)*** [0.128]***
PTA	0.025 (0.046) [0.055]	0.118 (0.041)*** [0.058]**		0.103 (0.041)** [0.056]*		0.106 (0.041)*** [0.060] *	
PTAXbenMdev			0.192 (0.064)*** [0.105]*		0.217 (0.065)*** [0.110]**		0.190 (0.066)*** [0.114]*
PTAXdevMben			-0.097 (0.082) [0.160]		0.004 (0.088) [0.163]		0.039 (0.088) [0.156]
PTAXbenMben			-0.073 (0.068) [0.101]		-0.008 (0.067) [0.099]		-0.088 (0.067) [0.099]
PTAXdevMdev			0.335 (0.069)*** [0.127]***		0.252 (0.074)*** [0.119]**		0.246 (0.074)*** [0.119]**
GATT/WTO	-0.150 (0.091)* [0.117]	0.207 (0.068)*** [0.107]*	0.206 (0.068)*** [0.107]*			0.107 (0.068)**	
GATTXbenMdev				0.266 (0.082)*** [0.139]*	0.253 (0.082)*** [0.140]**		0.252 (0.082)*** [0.139]*
GATTXdevMben				-0.049 (0.090) [0.141]	-0.036 (0.092) [0.140]		-0.011 (0.092) [0.135]
GATTXbenMben				0.210 (0.081)*** [0.138]	0.210 (0.081)*** [0.137]		0.208 (0.081)*** [0.136]
GATTXdevMdev				0.572 (0.112)*** [0.248]**	0.510 (0.124)*** [0.251]**		0.501 (0.124)*** [0.252]**
NRPTAXbenMdev	0.082 (0.051) [0.056]	0.084 (0.065) [0.0]	0.096 (0.045)** [0.066]	0.086 (0.044)* [0.066]	0.101 (0.045)** [0.066]		
NRPTAXdevMben	0.015 (0.045) [0.057]	0.050 (0.055) [0.099]	0.044 (0.057) [0.105]	0.112 (0.054)** [0.095]	0.104 (0.057)* [0.105]	0.020 (0.055) [0.100]	0.079 (0.056) [0.105]
NRPTAXbenMdev (up to 2008)						0.176 (0.051)*** [0.084]**	0.183 (0.052)*** [0.082]**
NRPTAXdevMben (from 2008)						-0.169 (0.058)*** [0.109]	-0.134 (0.059)** [0.105]
CYFE	Yes	Yes	Yes	Yes	Yes	Yes	Yes
CPFE	Yes	Yes	Yes	Yes	Yes	Yes	Yes
No observat.	269,146	269,146	269,146	269,146	269,146	212,384	212,384

Notes: We employ the *ppml_panel_sg* Stata command. The regressand in column 1 is the value of bilateral exports. The regressands in columns 2 to 7 are export shares of each country over total exports of that country. CU, PTA and NRPTA denote currency unions, preferential trade agreements and nonreciprocal preferential trade agreements, respectively. The suffixes Xben and Xdev in the name of the variables indicate exporter beneficiary and exporter developed, respectively. In a similar way, the suffixes Mben and Mdev in the name of the variables indicate importer beneficiary and importer developed, respectively. Robust standard errors clustered by country-pairs are in parentheses. In brackets we report multi-way clustered standard errors.

* significant at 10%

** significant at 5%

*** significant at 1%. CYFE indicates time-varying exporter and importer fixed effects. CPFE indicates country-pair fixed effects. Coefficient estimates for CYFE and CPFE are not reported for brevity.

[36] analyse the discrepancies between PPML and OLS estimators by Monte Carlo simulations. They point out that PPML uses deviations from levels of the trade flows with respect to their predictions, while the OLS involves log deviations. They demonstrate that PPML tend to put more weight on pairs of countries with large volumes of trade, which affects the results. Therefore, following Mayer et al. (2018) in the remaining columns of Table 3 we run PPML on export shares (bilateral exports divided by total exports) rather than export flows. That is, we estimate Eq (3) with PPML:

$$\left(\frac{X_{ijt}}{X_{it}}\right)=\exp(\beta_{1}CU_{ijt}+\beta_{2}PTA_{ijt}+\beta_{3}GATT/WTO_{ijt}+\beta_{4}NRPTAXbenMdev_{ijt}+\beta_{5}NRPTAXdevMben_{ijt}+\eta_{ij}+\chi_{it}+\lambda_{jt})+u_{ijt}$$ (3)

This will be our preferred specification because, unlike OLS, it deals with econometric problems resulting from heteroscedasticity and zeros but gives less weight to large flows in levels, as OLS does, since it works with export shares for a given exporter. A similar solution was advanced by [41]. As point out by [36], Sebastian Sotelo proved in unpublished notes that the multinomial PML can be estimated by applying the Poisson command to the market share variable along with country-specific fixed effects.

In line with Mayer et al. (2018), the results for the model with export shares as the dependent variable are more proximate to those found with OLS, when we use country-pair clustered standard errors. In particular, PPML estimates reported in column 2 reveal positive and statistically significant effects for CUs, PTAs, GATT/WTO and NRPTAs (in the direction in which the trade preferences are given). However, using multi-way rather country-pair clustering has particularly relevant consequences in this case. Apart from the fall in the level of statistical significance for the PTA and GATT dummies (which in any case remain statistically significant at the 5 and 10 per cent level, respectively) the effect for the nonreciprocal agreements vanishes.

Columns 3 to 5 present the results when we disaggregate alternatively the PTA dummy, the GATT/WTO dummy or both according to the group which each trading partner belongs to. Two comments are in order. First, in all the cases and regardless the type of standard errors considered, the results show a positive and statistically significant effect (at least at the 10 per cent level) for the dummies of the reciprocal agreements that capture the impact on exports when the developed countries are the destination markets. Second, the five statistically significant coefficient for nonreciprocal agreements in columns 3 to 5 when standard errors are country-pair clustered loss the statistical significance when standard errors are multi-way clustered.

Finally, columns 6 and 7 present the results when we again split the NRPTA dummy for exports from beneficiary countries to developed countries into two dummies in order to compare the results of the period before and after 2008. Curiously, the dummy leaving out the last years of the sample is positive and statistically significant (at least at the 5 per cent level) even when the standard errors are multi-way clustered. However, the estimated coefficient for the dummy from 2008 is never positive giving additional support to the argument of erosion of preferences in the last years.

## 5. Conclusions

The increase of exports from developing countries has long been considered an essential element to promote economic development. Over the last five decades, developed countries have provided preferential access to their markets to developing countries through nonreciprocal trade agreements. Moreover, developing countries have also participated in reciprocal trade agreements (bilateral, plurilateral or multilateral). in this paper we re-examine comparatively the effect of reciprocal and nonreciprocal trade agreements on exports from developing countries to developed countries but also on exports from the opposite direction.

The evidence across specifications is unstable. The results for the log-linear version of the gravity equation with OLS (including time-varying exporter and importer fixed effects as well as time-invariant country-pair fixed effects) suggest that both reciprocal and nonreciprocal trade agreements have had an economically and statistically significant impact regardless of the direction of the trade flows. However, this estimator may lead to biased results since it does not tackle the issues related to heteroskedasticity and zeros. Once we deal with these issues with the PPML estimator the picture that emerges is very different. We find the apparently implausible result that no economic integration agreement has had an effect on export flows. Given these discrepancies and following other authors we choose as our preferred specification a PPML estimation that uses export shares as the dependent variable and in which standard errors are multi-way clustered. According to this specification for the whole sample period only reciprocal agreements have had a positive effect in trade flows between developed and developing countries and only when the exporter is the developing country. For data up to 2008, there is evidence of a positive impact of the NRPTA on exports from beneficiary countries to the developed countries that vanishes in the last years. Finally, for exports from developed countries to beneficiary countries our preferred specification does not provide evidence of a significant effect in any case. In summary, the results of our preferred specification give additional support to the argument raised by critics of nonreciprocal preference regimes who consider that developing countries should abandon their reliance on one-way trade preferences in favor of reciprocal agreements.

## Supporting information

S1 Appendix (DOCX)

## References

[1] Ornelas E. Special and Differential Treatment for Developing Countries In: Bagwell K, Staiger RW, editors. Handbook of Commercial Policy, vol. 1B, Elsvier: Amsterdam 2016.

[2] Whalley J. Special and differential treatment in the millennium round. The World Economy. 1999; 22: 1065–1093.

[3] Whalley J. Non-discriminatory discrimination: special and differential treatment under the GATT for developing countries. Economic Journal. 1990; 100:1318–1328.

[4] Panagariya A. EU preferential trade arrangements and developing countries. The World Economy. 2002; 25:1415–1432.

[5] Özden C, Reinhardt E. The perversity of preferences: GSP and developing country trade policies, 1976–2000. Journal of Development Economics. 2005; 78: 1–21.

[6] Baier SL, Bergstrand JH. Do free trade agreements actually increase members' international trade? Journal of International Economics. 2007; 71:72–95.

[7] Behar A, Cirera-i-Crivillé L. Does it matter who you sign with? Comparing the impacts of north-south and south-south trade agreements on bilateral trade. Review of International Economics. 2013; 21:765–782.

[8] Herz B, Wagner M. The dark side of the Generalized System of Preferences. Review of International Economics. 2011; 19: 763–775.

[9] Gil-Pareja S, Llorca-Vivero R, Martínez-Serrano JA. Do nonreciprocal preferential trade agreements increase beneficiaries’ exports? Journal of Development Economics. 2014; 107: 291–304.

[10] Gil-Pareja S, Llorca-Vivero R, Martínez-Serrano JA. The effect of nonreciprocal preferential trade agreements on benefactors’ exports. Empirical Economics. 2017; 52: 143–154.

[11] Baier SL, Bergstrand JH, Feng M. Economic integration agreements and the margins of international trade. Journal of International Economics. 2014; 339–350.

[12] Klasen S, Martínez-Zarzoso I, Nowak-Lehmann D F, Brückner M. Does the designationof least developed country status promote exports? Discussion Papers, Ibero-America Institute for Economic Research N° 235. 2018.

[13] Ornelas E, Ritel M. The not-so-generalized effects of the Generalized System of Preferences. CEPR Discussion Paper DP 13208. 2018.

[14] Tinbergen J (1962) Shaping the World Economy. Twentieth Century Fund, New York.

[15] Pöyhönen P. A tentative model for the volume of trade between countries. Weltwirtschaftliches Arhiv. 1963; 90: 92–100.

[16] Larch M, Wanner J, Yotov Y, Zylkin T. The currency Union Effect: A PPML re-assessment with high-dimensional fixed effects. Drexel University Working Paper 2017–07. 2017

[17] Anderson JE, van Wincoop E. Gravity with Gravitas: A Solution to the Border Puzzle. American Economic Review. 2003; 93: 170–192.

[18] Baier SL, Bergstrand JH. On the endogeneity of international trade flows and free trade agreements. Available from: https://www3.nd.edu/~jbergstr/Working_Papers/EndogeneityAug2002.pdf

[19] Ch Magee. Endogenous preferential trade agreements: an empirical analysis. Contributions to Economic Analysis and policy. 2 (1) Berkeley Electronic Press. 2003.

[20] Baier L, Bergstrand JH, Egger P, McLaughlin P. Do economic integration agreements actually work? Issues in understanding the causes and consequences of growth of regionalism. The World Economy. 2008; 31:461–497.

[21] Gil-Pareja S, Llorca-Vivero R, Martínez-Serrano JA. Assessing the enlargement and deepening of the European Union. The World Economy. 2008a; 31: 1253–1272.

[22] Gil-Pareja S, Llorca-Vivero R, Martínez-Serrano JA. Trade effects of monetary unions: Evidence from OECD countries. European Economic Review. 2008b; 52: 733–755.

[23] Eicher TS, Henn Ch. In search of WTO trade effects: Preferential trade agreements promote trade strongly, but unevenly. Journal of International Economics. 2011a; 83: 137–153.

[24] Eicher TS, Henn Ch. One money, one market–A revisited benchmark. Review of International Economics. 2011b; 19: 419–435.

[25] Fugazza M, Nicita A. The direct and relative effects of preferential market access. Journal of International Economics. 2013; 89:357–368.

[26] Dutt P, Mihov I, Van Zandt T. The effect of WTO on the extensive and the intensive margins of trade. Journal of International Economics. 2013; 91:204–219.

[27] Kohl T. The WTO effect on trade: What you give is what you get In Christensen B J, Kowalczyk C (Eds.) Globalization: Strategies & Effects, Springer, Heidelberg 2015

[28] Gil-Pareja S, Llorca-Vivero R, Martínez-Serrano JA. A re-examination of the effect of GATT/WTO on trade. Open Economies Review. 2016; 27: 561–584.

[29] Silva JMCS, Tenreyro S. The log of gravity. The Review of Economics and Statistics. 2006; 88: 641–658.

[30] Liu X. GATT/WTO promotes trade strongly. Sample selection and model specification. Review of International Economics. 2009; 17:428–46.

[31] Felbermayr G, Kohler W. Modelling the extensive margin of world trade: new evidence on GATT and WTO membership. The World Economy. 2010; 33:1430–1469.

[32] Herz B, Wagner M. The real impact of GATT/WTO- A generalized approach. The World Economy. 2011 34: 1014–1041.

[33] Egger P, Larch M. An assessment of the Europe agreements’ effects on bilateral trade, GDP, and welfare. European Economic Review. 2011; 55: 263–279.

[34] Bergstrand JH, Larch M, Yotov YV. Economic integration agreements, border effects, and distance elasticities in the gravity equation. European Economic Review. 2015; 78: 307–327.

[35] Head K, Mayer T, Ries J. The erosion of colonial trade linkages after independence. Journal of International Economics. 2010; 81: 1–14.

[36] Head K, Mayer T, Ries J. Gravity equations: workhorse, toolkit, and cookbook In Gopinath G. Helpman E and Rogoff K, editors. Handbook of international economics, Elsevier vol. 4; 2014: 131–196

[37] Subramanian A, Wei S-W. The WTO promotes trade, strongly but unevenly. Journal of International Economics. 2007; 72: 151–175.

[38] Kohl T. Do we really know that trade agreements increase trade? Review of World Economics. 2014; 150: 443–469.

[39] Egger P, Tarlea F. Multi-way clustering estimation of standard errors in gravity models. Economics Letters. 2015; 134: 144–147.

[40] Mayer T, Vicard V, Zignago S. The cost of non-Europe, revisited. CEPR Discussion Paper DP 12844. 2018.

[41] Eaton D, Kortum S, Sotelo S. International trade: linking micro and macro. Technical report, NBER. 2012.

