# Supplementary material for: Reciprocal vs nonreciprocal trade agreements: Which have been best to promote exports?
Source: PLoS One. 2019 Feb 4;14(2):e0210446. doi: 10.1371/journal.pone.0210446 (PMC6361425; doi:10.1371/journal.pone.0210446)
Supplement: S1 Appendix — (DOCX) [file pone.0210446.s001.docx]

S1 Appendix

Table A. Pairs with positive values.

|  | 1968 | 1980 | 1992 | 2004 | 2016 |
| --- | --- | --- | --- | --- | --- |
| CU | 188 | 102 | 132 | 265 | 465 |
| PTA | 256 | 538 | 1,132 | 3,240 | 5,405 |
| PTAXben | 189 | 335 | 821 | 2,097 | 3,216 |
| PTAXdev | 67 | 203 | 311 | 1,143 | 2,189 |
| PTAMben | 189 | 335 | 821 | 2,097 | 3,216 |
| PTAXdev | 67 | 203 | 311 | 1,143 | 2,189 |
| PTAXbenMdev | 5 | 21 | 33 | 379 | 1.233 |
| PTAXdevMben | 5 | 21 | 33 | 379 | 1,233 |
| PTAXbenMben | 184 | 314 | 788 | 1,718 | 1,983 |
| PTAXdevMdev | 62 | 182 | 278 | 764 | 956 |
| GATT/WTO | 4,970 | 6,320 | 9,702 | 18,906 | 23,256 |
| GATTXben | 3,290 | 4,424 | 7,350 | 14,248 | 17,936 |
| GATTXdev | 1,680 | 1,896 | 2,352 | 4,658 | 5,320 |
| GATTMben | 3,290 | 4,424 | 7,350 | 14,248 | 17,936 |
| GATTMdev | 1,680 | 1,896 | 2,352 | 4,658 | 5,320 |
| GATTXbenMdev | 1,128 | 1,344 | 1,800 | 3,536 | 4,130 |
| GATTXdevMben | 1,128 | 1,344 | 1,800 | 3,536 | 4,130 |
| GATTXbenMben | 2,162 | 3,080 | 5,550 | 10,712 | 13,806 |
| GATTXdevMdev | 552 | 552 | 552 | 1,122 | 1,190 |
| NRPTAXbenMdev | 90 | 2,027 | 2,922 | 3,939 | 3,115 |
| NRPTAXdevMben | 90 | 2,027 | 2,922 | 3,939 | 3,115 |
| No observations | 33,124 | 33,124 | 33,124 | 33,124 | 33,124 |

Note: The variables are binary dummies. CU, PTA and NRPTA denote currency unions, preferential trade agreements and nonreciprocal preferential trade agreements, respectively. The suffixes Xben and Xdev in the name of the variables indicate exporter beneficiary and exporter developed, respectively. In a similar way, the suffixes Mben and Mdev in the name of the variables indicate importer beneficiary and importer developed, respectively.

Table B. Pairs with simultaneous membership in GATT/WTO and CU, PTA and NRPTA

|  | 1968 | 1980 | 1992 | 2004 | 2016 |
| --- | --- | --- | --- | --- | --- |
| CU | 58 | 34 | 60 | 236 | 432 |
| PTA | 94 | 258 | 638 | 2,402 | 4,477 |
| NRPTA | 70 | 960 | 1,501 | 2,762 | 2,537 |
| No observations | 33,124 | 33,124 | 33,124 | 33,124 | 33,124 |

Note: The variables are binary dummies
